# Supplementary material for: Trace element catalyses mineral replacement reactions and facilitates ore formation
Source: Nat Commun. 2021 Mar 2;12:1388. doi: 10.1038/s41467-021-21684-5 (PMC7925538; doi:10.1038/s41467-021-21684-5)
Supplement: Supplementary file 1 — Supplementary Material [file 41467_2021_21684_MOESM1_ESM.pdf]

Supplementary materials for

Trace element catalyses mineral replacement reactions and facilitates ore formation

Yanlu Xing[1,2,*], Joël Brugger[1,*], Barbara Etschmann[1], Andrew G. Tomkins[1], Andrew J. Frierdich[1], and Xiya Fang[3]

*Corresponding author. Email: yanluxxing@gmail.com; joel.brugger@monash.edu

Table 1 Summary of reaction conditions in batch Teflon reactors

Run	T (ºC)	pH	S/F (g/L)	Oxidant	NaCl/ M	Filling gas	Time /days	REE (ppm)	REE	Products
MH38	200	4	Acetate acid buffer		0.5	N_2	8	250.6	La	hem 23.4, mag 76.6
MH39	200	4	Acetate acid buffer		0.5	N_2	8	203.1	Ce	hem 54.1, mag 45.9
MH40	200	4	Acetate acid buffer		0.5	N_2	8	168.5	Nd	hem 22.7, mag 77.3
MH41	200	4	Acetate acid buffer	0.2g H_2O_2	0.5	N_2	8	202.0	La	hem 15.8, mag 84.2
MH42	200	4	Acetate acid buffer	0.2g H_2O_2	0.5	N_2	8	237.0	Ce	hem 11.5, mag 88.5
MH43	200	4	Acetate acid buffer	0.2g H_2O_2	0.5	N_2	8	180.0	Nd	hem 12.1, mag 87.9
MH44	200	4	Acetate acid buffer		0.5	N_2	14	255.8	Ce	hem 60.5, mag 39.6
MH45	200	4	Acetate acid buffer		0.5	N_2	14	321.7	La	hem 22.8, mag 77.3
MH46	200	4	Acetate acid buffer	0.2g H_2O_2	0.5	Air	14	267.1	Ce	hem 12.7, mag 87.3

NOTE: The numbers in the Products indicate the percentage of hematite (hem) and magnetite (mag) in the products.

Textures for samples reacted in oxidative solutions

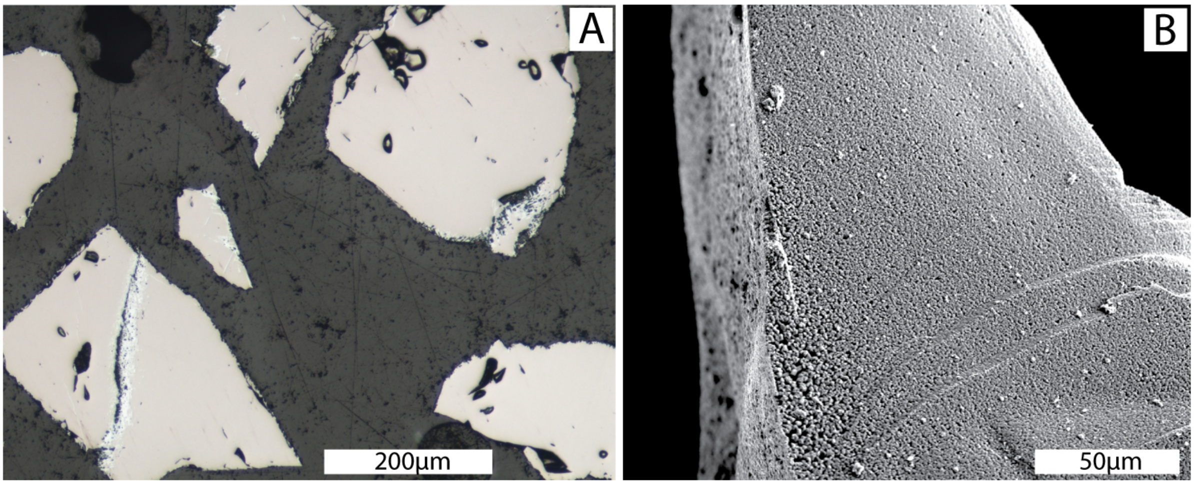

Fig. 1. Optical microscopy and SE image of samples reacted in oxidative solution

(A) Little replacement of magnetite replacement along the rim or cracks. (B) Surface of the grain is smooth, with little porosity developed.

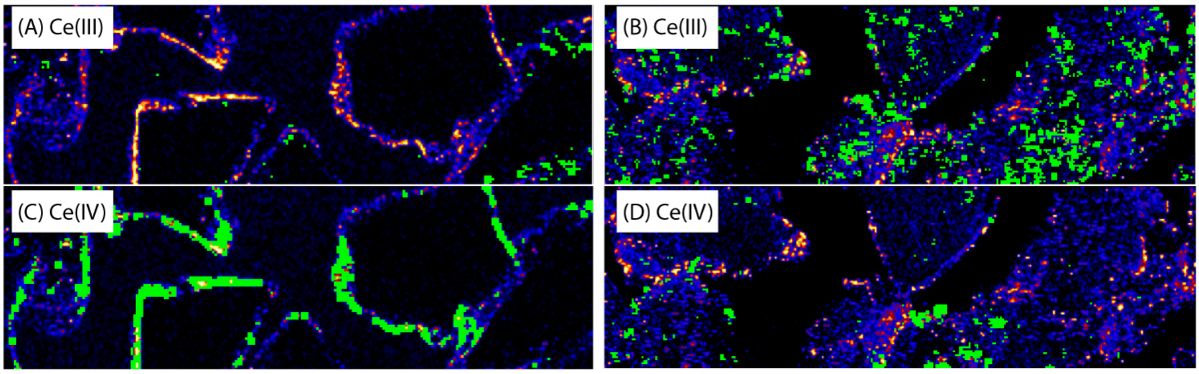

Fig. 2. XFM mapping for Ce(III) and Ce(IV) of samples from oxidative (A, C) and non-oxidative(B, D) solutions

(A) Ce(III) and (C) Ce(IV) distribution for sample run under oxidative condition. (B) Ce(III) and (D) Ce(IV) distribution for sample run under non-oxidative condition.

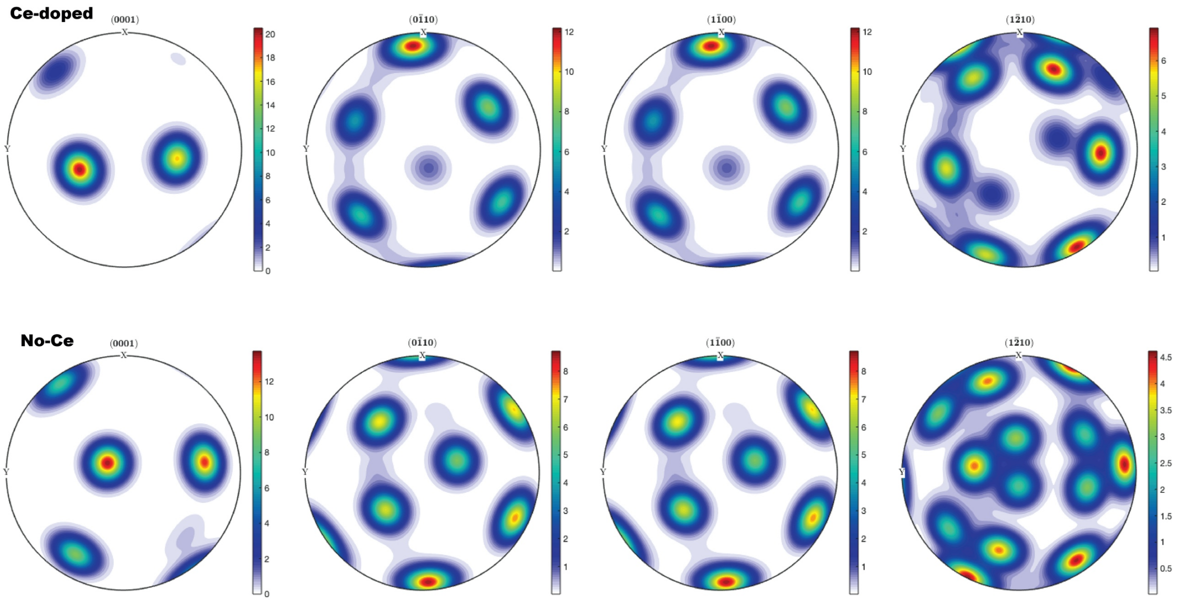

Fig. 3. Pole figure of hematite from both Ce-doped solution and Ce-free solution

These pole figures are plotted from EBSD mapping data. In both scenarios, the transformation obeys the topotaxial and epitaxial relationship that are well described hematite replacing magnetite, with $\{111\}_{mt}$ parallel to $\{0001\}_{hm}$; this also results in 3 orientations $\{0\bar{1}10\}\{\bar{1}100\}\{1\bar{2}10\}$ of hematite via replacement of a single crystal magnetite. For each orientation, hematite grains are more concentrated in Ce-bearing solution compared to Ce-free solution. mt: magnetite; hm: hematite.

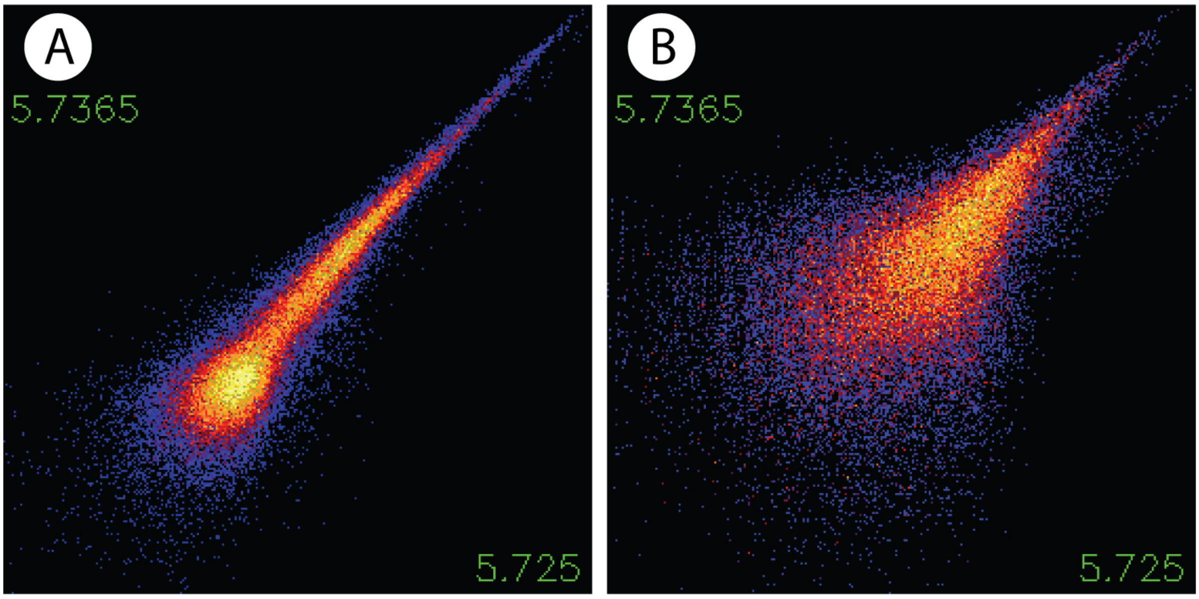

Fig. 4. XANES energy distribution of Ce for samples from oxidative and non-oxidative solutions

(A) Sample reacted in oxidative solution. The mapping shows a slim, single tail with less low energy points, indicating that the energy for Ce is concentrated and Ce^{4+} is the predominant species. (B) Sample reacted in non-oxidative solution. Mapping shows two tails and more low energy points, indicating that Ce distribution is more separated, compared to samples reacted in oxidative solutions; Ce^{3+} and Ce^{4+} are both important while Ce^{3+} is the predominant.

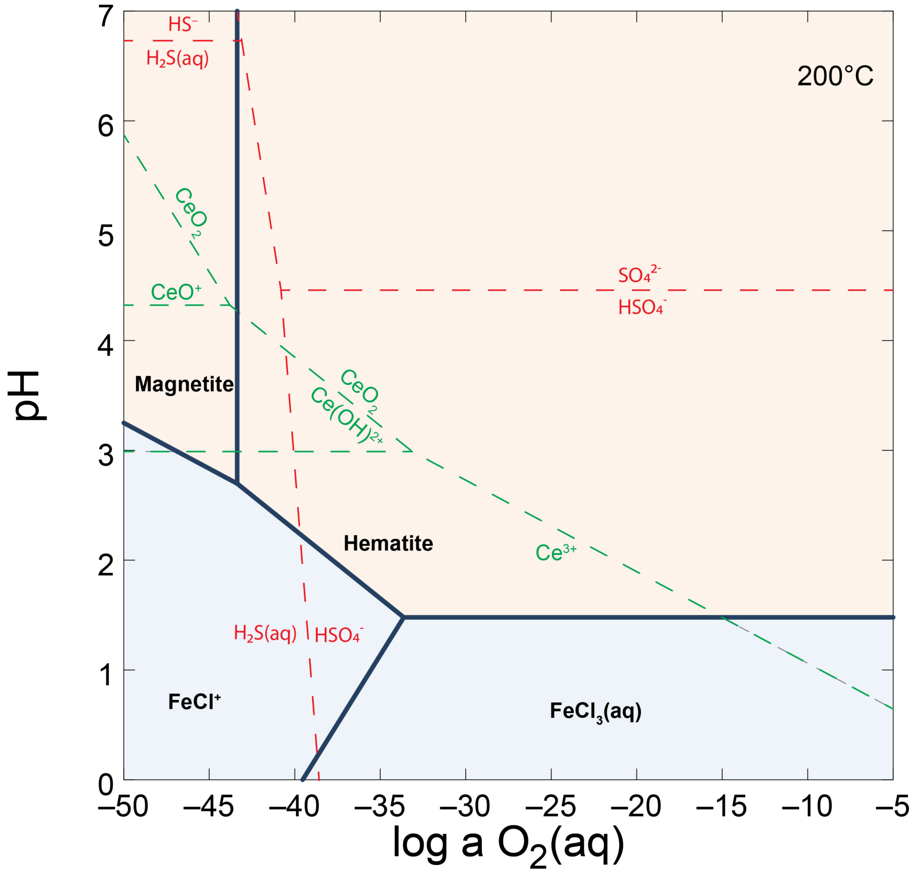

Fig. 5. pH-fO_2 activity diagram for predominant Fe speciation at 200 ºC, saturation pressure.

a(Ce^{3+}) = $10^{-3.5}$, a(SO_4^{2-}) = 10^{-5}, a(Cl) = 1. At pH~4, Ce(III) and Ce(IV) interconversion can happen in response to subtle change of pH and fO_2 during magnetite to hematite transformation reaction. CeO_2 is stable solid phase at SO_4^{2-}/HSO_4^- regime under less acidic conditions.
